# A novel synthetic quantification standard including virus and internal report targets: application for the detection and quantification of emerging begomoviruses on tomato

**DOI:** 10.1186/1743-422X-8-389

**Published:** 2011-08-05

**Authors:** Frédéric Péréfarres, Murielle Hoareau, Frédéric Chiroleu, Bernard Reynaud, Jacques Dintinger, Jean-Michel Lett

**Affiliations:** 1 CIRAD, UMR PVBMT CIRAD-Université de la Réunion, Pôle de protection des plantes, 7 chemin de l'IRAT, 97410 Saint Pierre, Ile de la Réunion, France

## Abstract

**Background:**

Begomovirus is a genus of phytopathogenic single-stranded DNA viruses, transmitted by the whitefly *Bemisia tabaci*. This genus includes emerging and economically significant viruses such as those associated with Tomato Yellow Leaf Curl Disease, for which diagnostic tools are needed to prevent dispersion and new introductions. Five real-time PCRs with an internal tomato reporter gene were developed for accurate detection and quantification of monopartite begomoviruses, including two strains of the *Tomato yellow leaf curl virus *(TYLCV; Mld and IL strains), the Tomato leaf curl Comoros virus-like viruses (ToLCKMV-like viruses) and the two molecules of the bipartite *Potato yellow mosaic virus*. These diagnostic tools have a unique standard quantification, comprising the targeted viral and internal report amplicons. These duplex real-time PCRs were applied to artificially inoculated plants to monitor and compare their viral development.

**Results:**

Real-time PCRs were optimized for accurate detection and quantification over a range of 2 × 10^9 ^to 2 × 10^3 ^copies of genomic viral DNA/μL for TYLCV-Mld, TYLCV-IL and PYMV-B and 2 × 10^8 ^to 2 × 10^3 ^copies of genomic viral DNA/μL for PYMV-A and ToLCKMV-like viruses. These real-time PCRs were applied to artificially inoculated plants and viral loads were compared at 10, 20 and 30 days post-inoculation. Different patterns of viral accumulation were observed between the bipartite and the monopartite begomoviruses. Interestingly, PYMV accumulated more viral DNA at each date for both genomic components compared to all the monopartite viruses. Also, PYMV reached its highest viral load at 10 dpi contrary to the other viruses (20 dpi). The accumulation kinetics of the two strains of emergent TYLCV differed from the ToLCKMV-like viruses in the higher quantities of viral DNA produced in the early phase of the infection and in the shorter time to reach this peak viral load.

**Conclusions:**

To detect and quantify a wide range of begomoviruses, five duplex real-time PCRs were developed in association with a novel strategy for the quantification standard. These assays should be of a great interest for breeding programs and epidemiological surveys to monitor viral populations.

## Background

The genus *Begomovirus *(family *Geminiviridae*) is a group of emerging phytopathogenic viruses transmitted by the whitefly *Bemisia tabaci *in a circulative permanent manner [1]. Begomoviruses cause severe diseases in a wide variety of plant species including many of considerable agricultural importance in tropical and sub-tropical areas [2]. Begomovirus genomes consist of monopartite or bipartite components of circular single strand DNA (ssDNA) [3]. The bipartite begomovirus genome is composed of two similar sized DNA molecules named DNA-A and DNA-B that share little sequence identity except for a 200nt region with at least 85% identity known as common region (CR) [4]. DNA-A component contains virus-encoded functions required for replication, transcription and encapsidation while the DNA-B component encodes proteins involved in intra- and inter-cellular viral movement [5] and symptom development [6]. The monopartite begomovirus genome is homologous to the DNA-A component of the bipartite with an additional viral-sense ORF, the precoat or V2, implicated in viral movement and pathogenicity [7]. Whereas in monopartite begomoviruses the single DNA-A like component is sufficient for infection, for bipartite begomoviruses, both DNA components are necessary for a systemic symptomatic infection and thus must be co-transmitted into a target cell to initiate the infection [8].

Based on their genome organization, their genetic diversity, and their geographical distribution, begomoviruses have been divided into two groups: Old World (Africa, Asia, Australia and Europe) and New World (America) begomoviruses [9]. Although no native monopartite begomovirus from the New World has been described, the *Tomato yellow leaf curl virus*, (TYLCV), a monopartite begomovirus, was accidentally introduced into America [10,11], and is now widespread in North America, Central America and the Caribbean. Its global spread represents one of the most serious threats to worldwide tomato production, including temperate, sub-tropical and tropical areas [12]. In addition to TYLCV, a wide range of begomoviruses [13] are associated with the tomato yellow leaf curl disease and sanitation measures are essential to prevent further introductions and dispersion of these devastating viruses.

The use of real-time PCR to detect and quantify RNA and DNA viruses from plants and/or insects has become particularly appealing due to both its speed and greater accuracy compared with serological or end-point PCR [14-17].

Most notably, duplex real-time PCR, with a plant gene as internal control, allows normalisation between samples. This procedure removes any sampling, extraction or amplification bias that could hamper the analyses and permits direct comparisons between independent samples and avoids false negatives.

In this paper, we describe the development of five duplex real-time PCRs for the detection and quantification of a wide range of begomoviruses responsible for the tomato yellow leaf curl disease in French overseas departments (Martinique and Guadeloupe [18], Reunion [19,20] and Mayotte [21-23]). These diagnostic tools are coupled with an original strategy: a unique quantification standard comprising the viral and internal report targets. All five duplex PCRs were applied to artificially inoculated plants to monitor and compare the viral accumulation of a bipartite begomovirus (*Potato yellow mosaic virus*, PYMV) and monopartite begomoviruses including two strains of one of the most emergent plant viruses (TYLCV) as well as species restricted to the Comoros archipelago, collectively recorded as Tomato leaf curl Comoros virus-like viruses (ToLCKMV-like viruses).

## Methods

### Design of primers and probes

Alignments of complete sequences of TYLCV-IL (n = 41), TYLCV-Mld (n = 16), PYMV (n = 3, DNA-A and DNA-B) and ToLCKMV-like viruses (ToLCKMV, ToLCYTV, ToLCMohV n = 4) were performed using the Clustal-W subalignment tool [24] available in MEGA 4 [25] (the different isolates used and the sequence alignments are presented in Additional File 1 and 2). For the internal control, the sequence of the nuclear-encoded large subunit ribosomal RNA gene (*Solanum lycopersicum 25S ribosomal RNA *gene (Sl25S; GenBank: X13557) was selected. Primers and MGB-probes were designed using the Primer Express Software for real-time PCR version 3.0 (Applied Biosystems). All primers and probes were purchased commercially (Applied Biosystems, Foster City, USA). The sequences, the ORF targeted and the labels of primers and probes developed in this study are listed in Table 1.

**Table 1:** Primers and probes developed and used in this study

Primers/Probes	Sequence 5'-3'	Label	Targeted ORF
F-Sl-25S	CGCCCGGTCGTACTCATAA	none	
R-Sl-25S	TCCATCGACCAGAGGCTGTT	none	NA
P-Sl-25S	CGCATCAGGTCTCCA	VIC	

PYMV-A-138-F	GCCTCTTGGCCCACTCTCTT	none	
PYMV-A-201-R	GCCATTGAACGCCATGGA	none	CP
PYMV-A-160-PMGB	ACTCAAAATGCCTAAGCG	FAM	

PYMV-B-1356-F	TGCAGACTCTCCCGGATCTAG	none	
PYMV-B-1415-R	CATCCGTATCGAGATCTGCAAA	none	MP
PYMV-B-1378-PMGB	ACGCTTGCTCCCAGC	FAM	

TYLCV-Mld-2186-F	CCTCTGACTTACTGCCTGAGTTAAGA	none	
TYLCV-Mld-2246-R	GGTCAGCAGTCAGCCAATGA	none	C4
TYLCV-Mld-2213-PMGB	CTGCGGCGTAAGC	FAM	

TYLCV-IL-2180-F	TGAGGGCCTCGGATTTATTG	none	
TYLCV-IL-2241-R	CAATCTGCCAACGACGCATA	none	C4
TYLCV-IL-2201-PMGB	CTGAATTGAGTGCTTCGG	FAM	

ToLCKMV-303-F	AGCGACCCGCCGATATAAT	none	
ToLCKMV-361-R	TTCAGTCTCCGACGCACCTT	none	CP
ToLCKMV-323-PMGB	ATTTCCACGCCCGCCT	FAM	

### Construction of the quantification standard into plasmid vector

**Figure 1:**
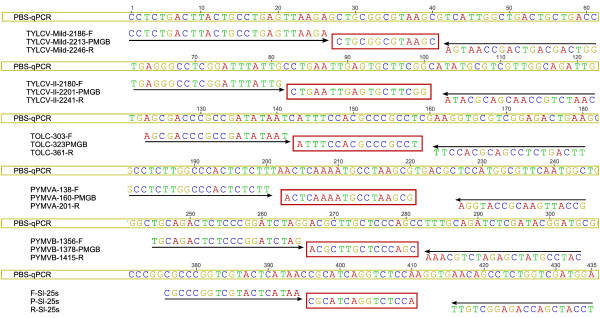
**Presentation of the 435 bp sequence synthetic quantification standard**. Alignment of qPCR targets of TYLCV-Mld, TYLCV-IL, ToLCKMV-like, PYMV (DNA-A and DNA-B), tomato internal control *Solanum lycopersicum 25S ribosomal RNA *gene (Sl25S; GenBank: X13557) with Taqman-MGB probes and forward and reverse primers.

The original feature of the assay was the construction of a quantification standard comprising the five viral and the internal report targets in a single plasmid vector (Figure 1) (see Additional File 3 for a schematic representation). The amplicons targeted by the different primer/probe systems were synthesized and cloned into *Sma*I-digested pBluescript II SK using Epoch Biolabs Inc. facilities (Missouri city, TX, USA). *Escherichia coli *strain JM-109 (Promega, Paris, France) cells were transformed with this plasmid. Recombinant plasmid DNA was isolated from bacteria with the Plasmid MiniPrep Spin Kit (Qiagen, Courtaboeuf, France) according to the manufacturer's instructions and quantified with the NanoDrop 8000 spectrophotometer (ThermoFisher Scientific, Courtaboeuf, France). The extracted plasmids were then serially diluted from 10^9 ^to 10^3 ^copies per μL in 10-fold steps, aliquoted and frozen before use as standards in each real-time PCR run. Standard curves were obtained by linear regression analysis of the threshold cycle (Ct) value of each of the two standard-dilution replicates over the log of the total amount of DNA. The Cts were automatically calculated by the StepOne Software v2.0 (Applied Biosystems, Courtaboeuf, France). The PCR efficiency

(E) was calculated as follows:

where a slope *s *= -3.322 represents an efficiency of 100%.

### Duplex real-time PCR optimizations

For the optimization of the five duplex real-time PCR assays, various primer concentrations (200-900 nM), probe concentrations (50-250 nM) and annealing-extension temperatures (60-65°C) were tested in a 15 μL reaction mix comprising 1× TaqMan universal PCR master mix (Applied Biosystems, Foster City, USA) and 2 μL DNA template. PCR reactions were carried out in the StepOnePlus real-time PCR system in fast optical 96-well reaction plates (Applied Biosystems, Courtaboeuf, France). Each sample was amplified in duplicate and a new aliquot of the standard was used in each run.

### Construction of agroinfectious clones of PYMV

Full-length DNA-A and DNA-B genomes of *Potato yellow mosaic virus-Tomato *[Guadeloupe:Tomato] (PYMV-To[GP:Tom], EMBL:AY120882/AY120883, [18]) were used for the construction of infectious clones in the binary vector pCambia0380 (Cambia, Camberra, Australia). A 1166 bp *Apa*I/*Nco*I digested fragment containing the intergenic region (IR) of the DNA-A was cloned to generate a 0.44-mer (pCambia0380-0.44). The full length monomer was cloned into *Apa*I digested pCambia0380-0.44 to generate a 1.44mer of PYMV-A. For the DNA-B, a 1367 bp *Eco*RI/*Bam*HI digested fragment containing the IR was cloned to generate a 0.54-mer (pCambia0380-0.54). The full length monomer was cloned into *Eco*RI digested pCambia0380-0.54 to generate a 1.54mer of PYMV-B. The orientation of the inserted genomes was checked by *Sac*I digestion. Recombinant plasmids were mobilized from *E. coli *strain JM-109 cells into *Agrobacterium tumefaciens *(strain C58) by triparental mating using *E. coli *HMB101 containing the plasmid helper pRK 2013 [26].

### Plant inoculation and total DNA extraction

Liquid culture of *A. tumefaciens *containing the agro-infectious clones of TYLCV-IL[RE4], TYLCV-Mld[RE], ToLCKMV-[YT:Dem:03] or PYMV-To[GP:Tom] (described respectively in [20,22] and this study) were grown for 14 h and adjusted to an OD_600 nm _of 1.0 before inoculation. For PYMV inoculation, equal amounts of *A. tumefaciens *containing PYMV-To[GP:Tom] (molecule A) and PYMV-To[GP:Tom] (molecule B) clones were mixed. Four sets of eight tomato plants *cv*. Farmer (Known-you Seed) were inoculated at the three-leaf stage by injecting 100 μL of *A. tumefaciens *culture into the stems (one set per virus).

Plants were then maintained in a complete random block design in an insect-free growth chamber at 26°C/24°C (day/night) with a 12-hour photoperiod. Virus accumulation was monitored using the duplex-real-time PCR developed in this study from the first true youngest leaf of each plant collected at 10, 20 and 30 days post-inoculation (dpi). Total DNA was extracted using the DNeasy Plant miniprep Kit (Qiagen, Courtaboeuf, France) according to the manufacturer's instructions. DNA was finally resuspended in 100 μL (two successive elutions of 50 μL) of ultrapure water and stored at -20°C until utilization.

Quantities of virus and internal report were calculated with the corresponding standard curves and results were expressed as the log of the ratio of the quantity of virus DNA to that of plant genomic DNA [27].

### Statistical analysis

The effects of the inoculated virus and the dpi were analysed on virus accumulation using an ANOVA procedure available in the R statistical software (R Development Core Team).

## Results

### Performance of the duplex-real-time PCR developed

Primer and TaqManMGB-probe concentrations for the duplex real-time PCR were first optimized to obtain the best efficiency in the larger linear dynamic range (data not shown). Selected conditions of the five duplex real-time PCR assays developed in this study are summarized in Table 2. All cycles begin with 2 min at 50°C then 10 min at 95°C follow by 40 two-step cycles comprising 15 s at 95°C and 1 min at the appropriate annealing-extension temperature (Table 2). In those conditions, no cross reaction was observed between the primers/probe system and non-targeted begomoviruses both in naturally field infected or artificially agro-inoculated plants (data not shown).

**Table 2:** Reaction conditions and assay performance of the five duplex real-time PCRs

Target	Virus		Internal report (ADN 25S)		T°m (°C)	Linear dynamic range (copies/uL)		PCR efficiency (%)	
			
	Primers	Probe	Primers	Probe		Virus	Internal report	Virus	Internal report
TYLCV-IL	650	150	500	100	63	2 × 10^9 ^to 2 × 10^3^	2 × 10^7^to 2 × 10^3^	96	108
TYLCV-Mld	250	200	750	150	64	2 × 10^9^to2 × 10^3^	2 × 10^9^to2 × 10^3^	103	107
PYMV-A	750	150	200	50	63	2 × 10^8^to2 × 10^3^	2 × 10^7^to2 × 10^3^	93	100
PYMV-B	900	150	200	50	63	2 × 10^9^to2 × 10^3^	2 × 10^9^to2 × 10^3^	97	100
ToLCKMV-like	750	150	300	50	62	2 × 10^8^to2 × 10^3^	2 × 10^7^to2 × 10^3^	95	104

**Figure 2:**
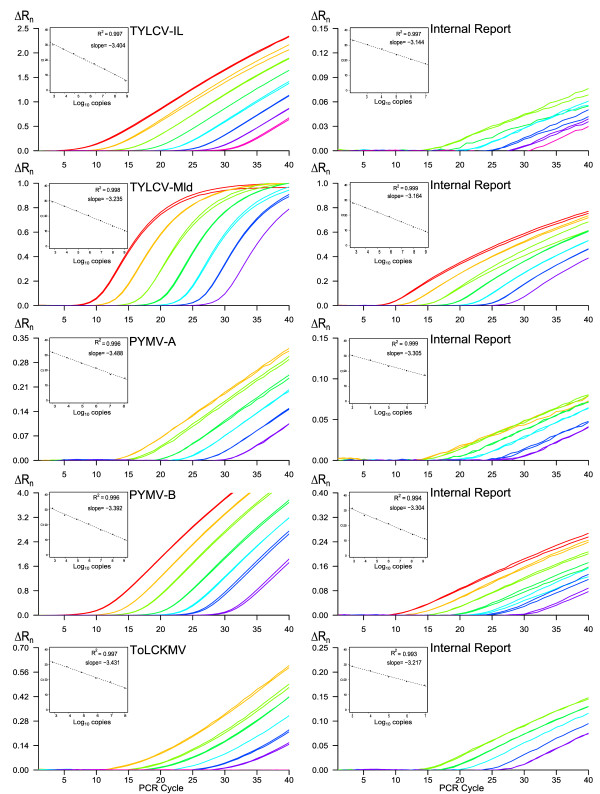
**Amplification plots and standard curves of the five duplex real-time PCRs**. Typical amplification plots for each viral target and the corresponding internal report in theirs linear dynamic ranges are represented. The corresponding standard curves are obtained by linear regression analysis of the threshold cycle (Ct) value of each of the two standard-dilution replicates over the log_10 _of the copies of DNA targets. The slope and the correlation coefficients are mentioned for each standard curve.

Typical amplification plots for the five duplex systems are shown in Figure 2. The corresponding standard curves had high correlation coefficients (R^2^>0.99), and calculated PCR efficiencies ranged from 93% to 108%. The linear dynamic ranges for the virus quantification were within the range of 2 × 10^9 ^to 2 × 10^3 ^copies/μL except for PYMV-A and ToLCKMV-like viruses, with a corresponding linear dynamic range of 2 × 10^8 ^to 2 × 10^3 ^copies/μL. The quantification of the internal report was possible in the range of 2 × 10^9 ^to 2 × 10^3 ^copies/μL for the TYLCV-Mld/Sl25S and PYMV-B/Sl25S duplexes, and 2 × 10^7 ^to 2 × 10^3 ^copies/μL for the TYLCV-IL/Sl25, PYMV-A/Sl25S and ToLCKMV-like viruses/Sl25S duplexes (Table 2).

### Virus accumulation in tomato plants

TYLCV-IL[RE4], TYLCV-Mld[RE], ToLCKMV-[YT:Dem:03] and PYMV-To[GP:Tom] were agro-inoculated in four sets of eight tomato plants to monitor the virus accumulation at 10, 20 and 30 dpi. All the plants inoculated with TYLCV-IL[RE4] and TYLCV-Mld[RE] produced typical yellow leaf curl and stunting symptoms between 10 and 20 dpi. All the plants inoculated with the PYMV-To[GP:Tom] developed typical symptoms of yellow mosaic on the leaves, curling and stunting between 13 and 20 dpi, confirming the pathogenicity of the partial tandem constructions. Six out of eight plants inoculated with ToLCKMV-[YT:Dem:03] became symptomatic between 15 and 25 dpi.

**Figure 3:**
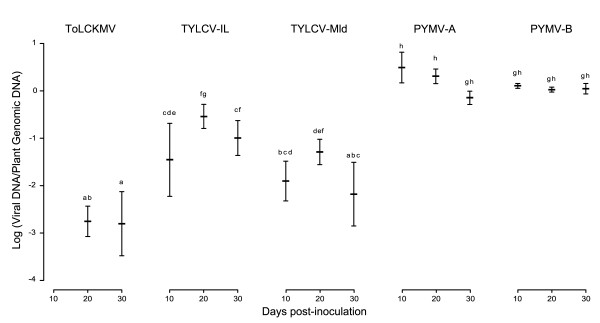
**Average virus accumulation of TYLCV-IL, TYLCV-Mld, ToLCKMV and PYMV (DNA-A and DNA-B) in tomato plants**. Data were obtained from 8 plants inoculated per virus. Vertical bars around each point represent the 95% confidence interval and the bars topped by the same letter (a to h) are not significantly different at p = 0.01 (Tuckey's HSD test). None of the inoculated plants with the ToLCKMV-[YT:Dem:03] were detected as infected at 10 dpi.

Effects of the virus inoculated, the dpi, and the interaction virus-dpi were highly significant on virus accumulation variations (p < 10^-8^, Fisher-Snedecor's test). Significant differences were found for viral accumulation between the different begomoviruses inoculated at each date considered (Figure 3). At 10 dpi, PYMV DNA-A and DNA-B accumulated on average 88-fold and 36-fold more viral DNA respectively than the TYLCV-IL (p < 10^-8 ^for PYMV DNA-A and DNA-B), and in average 248-fold and 101-fold more respectively than the TYLCV-Mld (p < 10^-8 ^for PYMV DNA-A and DNA-B). No difference was found between the genome A and B of the PYMV (p = 0.67) and between the two strains of the TYLCV (p = 0.29). None of the inoculated plants with the ToLCKMV-[YT:Dem:03] were detected as infected at 10 dpi. At 20 dpi, PYMV DNA-A accumulated on average 7-fold and 39-fold more than TYLCV-IL and TYLCV-Mld (p = 10^-3 ^and p < 10^-8 ^respectively) without any significant differences with PYMV DNA-B (p = 1). TYLCV-Mld accumulated on average 5-fold less than the IL strain (p = 8 × 10^-3^) but on average 29-fold more than the ToLCKMV (p = 4 × 10^-5^). At 30 dpi, PYMV DNA-A and DNA-B accumulated on average 7-fold and 11-fold more viral DNA than the TYLCV-IL respectively (p = 2 × 10^-3 ^and p = 6 × 10^-5 ^for PYMV DNA-A and DNA-B respectively) without any difference between the two molecules (p = 1). TYLCV-Mld accumulated on average 15-fold less viral DNA than the IL strain (p = 1 × 10^-5^) and there was no difference with ToLCKMV (p = 0.07). The asymptomatic plants inoculated with the ToLCKMV-[YT:Dem:03] remained virus-free or undetectable by the real-time PCR at 10, 20 and 30 dpi.

Significant differences were found in the accumulation kinetics of the begomoviruses considered. PYMV DNA-A and DNA-B reached their highest viral load at 10 dpi and maintained this level at 20 and 30 dpi (p = 1 for DNA-A and DNA-B for 20 and 30 dpi). TYLCV-IL reached its peak viral load at 20 dpi (p = 6 × 10^-4 ^with 10 dpi) and remained at this maximum viral load between 20 and 30 dpi (p = 0.31). TYLCV-Mld reached its peak viral load at 10 dpi and there was no difference between 10 and 20 dpi (p = 0.046). Between 20 and 30 dpi, the viral load decreased to the same level reached at 10 dpi (p = 1 × 10^-3 ^and p = 1 for 20 and 30 dpi and for 30 and 10 dpi respectively). In the case of ToLCKMV, at 20 dpi only four plants showed detectable levels of virus and reached their peak viral load. At 30 dpi, six plants were quantified, their viral loads were not different as compared to 20 dpi (p = 1).

## Discussion

During the last two decades, the spread of the highly polyphageous biotype B of *B. tabaci *has greatly contributed to the worldwide emergence of begomoviruses. These devastating viruses are one of the most important threats for tomato production in tropical and subtropical environments. Within the European Union, begomoviruses are listed on the EPPO A2 alert list and diagnostics tools are essential to prevent dispersions and new introductions. Several PCR-based methods [28,29] and real-time PCR [15,16] have been reported recently for the detection and the differentiation of strains and species of begomoviruses. We present here five real-time PCR assays including an internal report for the relative quantification of different begomoviruses in tomato plants. Those duplex real-time PCR assays are associated with a novel strategy for a unique quantification standard consisting in the cloning of both viral and internal report targets in the same plasmid. We developed and successfully applied these real-time PCR assays for the specific detection and quantification of a wide range of begomoviruses including the two emerging strains of TYLCV (IL and Mld strains), the PYMV and the ToLCKMV-like viruses.

As described previously in others studies [30,31], we used an internal report to validate and normalize the entire experiment including the processes of sampling, DNA extraction and DNA amplification. Following Mason et al. [15], we selected the *Solanum lycopersicum 25S ribosomal RNA *gene as internal report and we optimized the real-time PCR to amplify, in the same reaction, both viral and host DNA targets. The original feature of our assay was the design of a unique standard quantification comprising both the viral and the internal report targets. Recently, Lay et al [32] described a similar approach with the cloning of two Epstein-Barr virus targets in a single quantification standard. Here, we have conceived a quantification standard comprising not only the five viral targeted amplicons but also the internal report target. This approach is very useful to reduce the laborious stages of preparation of quantification standards containing known amounts of each target to a single step, and thus reduces the time and the cost of the whole assay.

The real-time PCR assays developed in this study were optimized to detect and quantify both the viral and host DNA in multiplex reactions respecting the MIQE guidelines [33]. PCR amplifications of the internal report cover six (TYLCV-IL, PYMV-A and ToLCKMV-like viruses) to seven (TYLCV-Mld and PYMV-B) orders of magnitude. Viral detection and quantification are possible in the range of 2 × 10^9 ^to 10^3 ^viral DNA copies/μL except for PYMV-A and ToLCKMV-like (2 × 10^8 ^to 10^3 ^viral DNA copies/μL). Those real-time PCR assays provide an accurate detection and quantification of the targeted viruses, with a higher detection limit than the ones previously described by others studies on RNA viruses [34,35] or DNA viruses [15] albeit of only a 10-fold. Advantageously, our real-time PCR assays are able to quantify both the host and viral DNA in a single run, making a direct normalisation of the quantification possible.

We successfully used the real-time PCR developed with experimentally inoculated plants to compare viral accumulation at 10, 20 and 30 dpi. These three successive viral quantifications, although unable to reflect the entire kinetics of viral accumulation, were sufficient to observe different patterns of viral accumulation between the bipartite and the monopartite begomoviruses and between the different strains and species of monopartite begomoviruses analysed.

The comparison of relative loads of viral DNA demonstrated that the bipartite PYMV accumulated more viral DNA than the two strains of TYLCV and ToLCKMV in tomato plants at each date considered. To our knowledge, it is the first demonstration of the higher viral load of a bipartite begomovirus than monopartite begomoviruses. The DNA-B component of begomoviruses encodes two viral proteins with essential functions in intra- and inter-cellular efficient movement [5] and can contribute to symptom production [6]. Although the origin of the DNA-B remains unclear [36], this component must provide selective advantages with enhanced viral fitness [36]. *A contrario *to TYLCV, the capacity of bipartite begomoviruses to escape from the phloem cells and infect the surrounding tissues could be a key element in this difference of the viral accumulation observed [7,37]. This wider tissue tropism gives the opportunity to infect more plant cells and may be the major determinant in our observed difference in viral accumulation. Our data revealed strong differences in the viral load between the bipartite and the monopartite begomoviruses at the leaf-level but the question of viral accumulation in a single infected cell remains open.

In the case of PYMV, interestingly, no difference was observed between the two genomic components accumulation at 10, 20 and 30 dpi with the higher viral loads reached at 10 dpi for both components. DNA-B of bipartite begomoviruses is necessary for viral infection, and so the two components must be co-transmitted to spread and induce systemic symptomatic infections [8] (for exception see [38]). Our data provide new insights into the replication of bipartite begomoviruses and suggest that both PYMV molecules accumulate at the same level from the early to the late phase of the infection, ensuring a further efficient transmission although we cannot exclude differential time to reach this peak viral load during the first ten days post-infection.

We also revealed differences in the patterns of viral accumulation between the two strains of TYLCV (Mld and IL strains). Experimental work using TYLCV, the monopartite ToLCV form Australia and TYLCCNV showed that the ORF C4 is implicated in viral movement [39], symptom development [40] and bypass defence mechanisms of the host [41,42]. Considering the recombinant nature of TYLCV-IL, which shares a common origin for a portion of its genome comprising the C4 ORF with ToLCV-Asian-like ancestors [43], we can hypothesize that the C4 protein of TYLCV-IL is in part responsible for the higher fitness observed compared to the Mld strain.

Finally, we revealed strong differences between the two strains of TYLCV and the ToLCKMV. The accumulation kinetics of these two species differed both in the quantity of viral DNA produced, and in the time to reach this peak viral load. Such differences could have strong epidemiological consequences increasing the probability for an insect vector to acquire a virus from a plant with higher viral load during a longer timeframe and thus contributing to their preferential dispersions. In this study, we compared two species of monopartite begomoviruses with very different impacts and areas of distribution. While the TYLCV is considered as one of the most emergent plant viruses and has succeeded in spreading worldwide [10], the ToLCKMV-like viruses are for now confined to the Comoros archipelago with a minor impact on local production [22]. Those two contrasted epidemiological profiles coincide with the strong differences in biological properties revealed by our study, such as the higher fitness. Our study thus suggests possible reasons for the successful spread of the emergent TYLCV, as compared to the indigenous and area-restricted ToLCKMV-like viruses.

## Conclusions

In this paper, we described an original real-time PCR strategy using a unique synthetic quantification standard comprising both viral and internal report targeted amplicons. The assays developed could be used to detect and quantify the four viruses studied in artificially inoculated plants. This approach is very useful in reducing the time and cost of the assays and could be extended to pathogen amplicons targeted by other real-time PCR assays. This strategy and the tools developed could be suitable and advisable for laboratories involved in plant certification or diagnosis.

## Competing interests

The authors declare that they have no competing interests.

## Authors' contributions

FP developed the assays and carried out all the DNA work. MH was involved in the agroinoculations and DNA extraction. FP and FC were involved in the statistical data analysis. FP and JML analyzed the data, prepared the manuscript and were involved in the design and conception of the study. JML, JD and BR secured funding for the project and provided ideas and comments during preparation of the manuscript. All authors have read and approved the final manuscript.

## Supplementary Material

Additional file 1 **Isolate, acronym and accessions numbers of the TYLCD-associated viruses used for sequences alignment and design of the primers and probes**. Click here for file

Additional file 2 **Alignments of the targeted isolates used to design primers and probes**. Taqman-MGB probes and forward and reverse primers are represented on each alignment. Click here for file

Additional file 3 **Schematic representation of the synthetic quantification standard**. Click here for file
