# Supplementary material for: A novel synthetic quantification standard including virus and internal report targets: application for the detection and quantification of emerging begomoviruses on tomato
Source: Virol J. 2011 Aug 5;8:389. doi: 10.1186/1743-422X-8-389 (PMC3175178; doi:10.1186/1743-422X-8-389)
Supplement: Additional file 2 — Alignments of the targeted isolates used to design primers and probes. Taqman-MGB probes and forward and reverse primers are represented on each alignment. [file 1743-422X-8-389-S2.PDF]

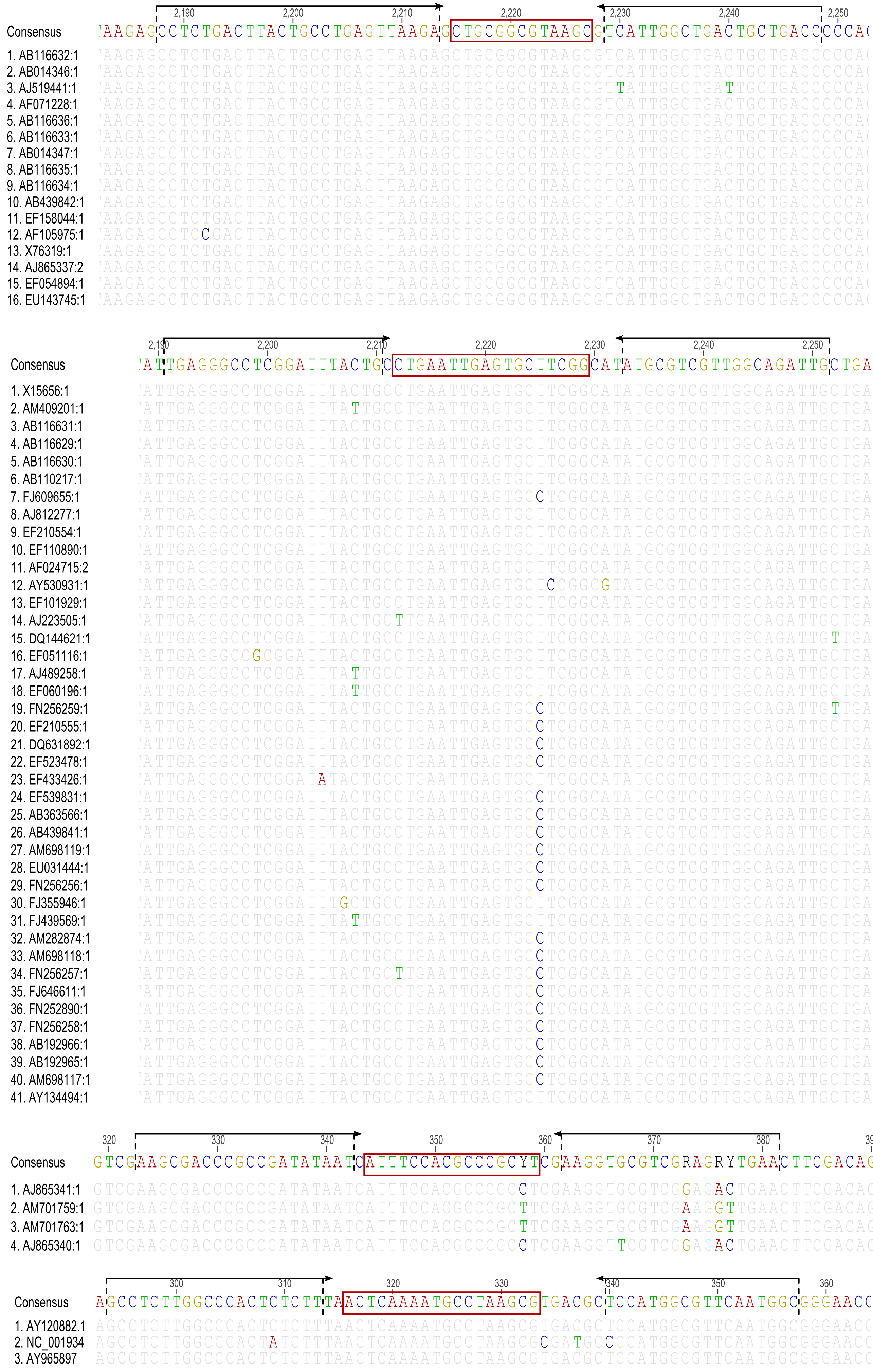

2,190 2,200 2,210 2,220 2,230 2,240 2,250
Consensus AAGAGCCTCTGACTTACTGCCTGAGTTAAGAGCTGCGGCGTAAGCGTCATTGGCTGACTGCTGACCCCCAC
1. AB116632:1
2. AB014346:1
3. AJ519441:1
4. AF071228:1
5. AB116636:1
6. AB116633:1
7. AB014347:1
8. AB116635:1
9. AB116634:1
10. AB439842:1
11. EF158044:1
12. AF105975:1
13. X76319:1
14. AJ865337:2
15. EF054894:1
16. EU143745:1
2,190 2,200 2,210 2,220 2,230 2,240 2,250
Consensus ATTGAGGGCCTCGGATTTACTGCCTGAATTGAGTGCTTCGGCATATGCGTCGTTGGCAGATTGCTGA
1. X15656:1
2. AM409201:1
3. AB116631:1
4. AB116629:1
5. AB116630:1
6. AB110217:1
7. FJ609655:1
8. AJ812277:1
9. EF210554:1
10. EF110890:1
11. AF024715:2
12. AY530931:1
13. EF101929:1
14. AJ223505:1
15. DQ144621:1
16. EF051116:1
17. AJ489258:1
18. EF060196:1
19. FN256259:1
20. EF210555:1
21. DQ631892:1
22. EF523478:1
23. EF433426:1
24. EF539831:1
25. AB363566:1
26. AB439841:1
27. AM698119:1
28. EU031444:1
29. FN256256:1
30. FJ355946:1
31. FJ439569:1
32. AM282874:1
33. AM698118:1
34. FN256257:1
35. FJ646611:1
36. FN252890:1
37. FN256258:1
38. AB192966:1
39. AB192965:1
40. AM698117:1
41. AY134494:1
320 330 340 350 360 370 380
Consensus GTCGAAGCGACCCGCCGATATAATCATTTCCACGCCCGCYTCGAAGGTGCGTCGRAGRYTGAACTTCGACAG
1. AJ865341:1
2. AM701759:1
3. AM701763:1
4. AJ865340:1
300 310 320 330 340 350 360
Consensus AGCCTCTTGGCCCACTCTCTTTAACTCAAAATGCCTAAGCGTGACGCTCCATGGCGTTCAATGGCGGGAACC
1. AY120882.1
2. NC_001934
3. AY965897
