# Supplementary figures and images for: A novel synthetic quantification standard including virus and internal report targets: application for the detection and quantification of emerging begomoviruses on tomato
Source: Virol J. 2011 Aug 5;8:389. doi: 10.1186/1743-422X-8-389 (PMC3175178; doi:10.1186/1743-422X-8-389)

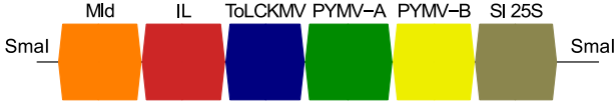

Supplement: Additional file 3 — Schematic representation of the synthetic quantification standard. [file 1743-422X-8-389-S3.PDF]
